# Reduced Mitochondrial Membrane Potential Is a Late Adaptation of *Trypanosoma brucei brucei* to Isometamidium Preceded by Mutations in the γ Subunit of the F_1_F_o_-ATPase

**DOI:** 10.1371/journal.pntd.0004791

**Published:** 2016-08-12

**Authors:** Anthonius A. Eze, Matthew K. Gould, Jane C. Munday, Daniel N. A. Tagoe, Valters Stelmanis, Achim Schnaufer, Harry P. De Koning

**Affiliations:** 1 Institute of Infection, Immunity and Inflammation, College of Medical, Veterinary and Life Sciences, University of Glasgow, Glasgow, United Kingdom; 2 Institute for Immunology and Infection Research and Centre for Immunity, Infection and Evolution, School of Biological Sciences, University of Edinburgh, Edinburgh, United Kingdom; 3 Wellcome Trust Centre for Molecular Parasitology, University of Glasgow, Glasgow, United Kingdom; Hunter College, CUNY, UNITED STATES

## Abstract

**Background:**

Isometamidium is the main prophylactic drug used to prevent the infection of livestock with trypanosomes that cause Animal African Trypanosomiasis. As well as the animal infective trypanosome species, livestock can also harbor the closely related human infective subspecies *T*. *b*. *gambiense* and *T*. *b*. *rhodesiense*. Resistance to isometamidium is a growing concern, as is cross-resistance to the diamidine drugs diminazene and pentamidine.

**Methodology/Principal Findings:**

Two isometamidium resistant *Trypanosoma brucei* clones were generated (ISMR1 and ISMR15), being 7270- and 16,000-fold resistant to isometamidium, respectively, which retained their ability to grow *in vitro* and establish an infection in mice. Considerable cross-resistance was shown to ethidium bromide and diminazene, with minor cross-resistance to pentamidine. The mitochondrial membrane potentials of both resistant cell lines were significantly reduced compared to the wild type. The net uptake rate of isometamidium was reduced 2-3-fold but isometamidium efflux was similar in wild-type and resistant lines. Fluorescence microscopy and PCR analysis revealed that ISMR1 and ISMR15 had completely lost their kinetoplast DNA (kDNA) and both lines carried a mutation in the nuclearly encoded γ subunit gene of F_1_ ATPase, truncating the protein by 22 amino acids. The mutation compensated for the loss of the kinetoplast in bloodstream forms, allowing near-normal growth, and conferred considerable resistance to isometamidium and ethidium as well as significant resistance to diminazene and pentamidine, when expressed in wild type trypanosomes. Subsequent exposure to either isometamidium or ethidium led to rapid loss of kDNA and a further increase in isometamidium resistance.

**Conclusions/Significance:**

Sub-lethal exposure to isometamidium gives rise to viable but highly resistant trypanosomes that, depending on sub-species, are infective to humans and cross-resistant to at least some diamidine drugs. The crucial mutation is in the F_1_ ATPase γ subunit, which allows loss of kDNA and results in a reduction of the mitochondrial membrane potential.

## Author Summary

Isometamidium is the only prophylactic treatment of Animal African Trypanosomiasis, a wasting disease of livestock and domestic animals in sub-Saharan Africa. Unfortunately resistance threatens the continued utility of this drug after decades of use. Not only does this disease have severe impacts on agriculture, but some subspecies of *Trypanosoma brucei* are human-infective as well (causing sleeping sickness) and there is concern that cross-resistance with trypanocides of the diamidine class could further undermine treatment of both veterinary and human infections. It is therefore essential to understand the mechanism of isometamidium resistance and the likelihood for cross-resistance with other first-line trypanocides. Here, we report that isometamidium resistance can be caused by a mutation in an important mitochondrial protein, the γ subunit of the F_1_ ATPase, and that this mutation alone is sufficient for high levels of resistance, cross-resistance to various drugs, and a strongly reduced mitochondrial membrane potential. This report will for the first time enable a structural assessment of isometamidium resistance genes in *T*. *brucei* spp.

## Introduction

*Trypanosoma brucei brucei*, *T*. *congolense* and *T*. *vivax* are the main parasites causing African animal trypanosomosis (AAT, also known as nagana) in much of sub-Saharan Africa, where they are transmitted by the tsetse fly. AAT affects most domestic animals, including bovines, equines, and pigs, and as such has a devastating effect on food production in the tsetse belt [1]. As there is no realistic prospect of a vaccine for African trypanosomiasis [2], and vector eradication is impracticable considering the size of the area affected, chemotherapy remains the only realistic option for livestock farmers in the region. However, only three treatments currently exist [3]: the diamidine diminazene aceturate (DA, Berenil), which is the standard treatment for AAT, the phenanthridine isometamidium chloride (ISM; Samorin), which is the standard prophylactic but can also be used therapeutically, and ethidium bromide (Homidium), which also has utility as prophylaxis against AAT but is mostly used therapeutically [4,5]. Although there are serious safety concerns about the therapeutic use of the carcinogenic ethidium, interest in the chemical and pharmacological properties of phenanthridines has recently increased, resulting in a multitude of analogs with, for example, preferential binding to either RNA or DNA, or functioning as DNA intercalator or minor groove binder [6].

All three treatments are under threat from drug resistance and indeed cross-resistance [7–11], critically endangering the ability to keep livestock in many areas. However, it remains very difficult to assess the true spread of trypanocide resistance throughout Africa, which requires the experimental infections of large numbers of cattle [12], because of a lack of reliable and well-validated genetic markers [13]. For DA, it was thought that in *T*. *congolense*, as in *T*. *brucei* species [14–16], DA is taken up by an AT1/P2 aminopurine transporter and that its loss is the cause of the observed resistance [17,18]. However, it was later established that the *T*. *congolense* equivalent to *TbAT1*, *TcoAT1*, did not in fact transport DA and the gene was renamed *TcoNT10* [19]; no other genetic marker has as yet been identified.

Ethidium is believed to kill trypanosomes in part by preventing the proper replication of mitochondrial DNA [20], which in trypanosomes forms a clearly defined structure within the mitochondrion called the kinetoplast. Genes encoded in kinetoplast DNA (kDNA) are normally required for maintenance of the mitochondrial membrane potential ΔΨm [21,22], which in turn is required for protein import and metabolite exchange. ATP production in bloodstream form *T*. *brucei* is thought to be generated exclusively by glycolysis, mostly localized in specialized glycosomes [23], and oxidative phosphorylation does not take place in these cells, with complexes II, III and IV being absent [24,25]. However, the mitochondrial Trypanosome Alternative Oxidase (TAO) is required for the regeneration of NAD^+^ produced during glycolysis [26]. Mitochondrial activities that are essential for the viability of bloodstream form *T*. *brucei* include the alternative oxidase [27], the glycine cleavage complex [28], acetyl-CoA production [29,30] and probably iron-sulfur cluster biosynthesis [31].

Nothing is currently known about the mechanism of ethidium resistance, although the frequently observed cross-resistance with ISM has led to speculation that their resistance mechanisms may be identical or at least overlap [5], and the mechanism of ISM resistance has been more extensively investigated than that of the other veterinary trypanocides. Similar to ethidium, an important part of ISM’s mode of action involves its effect on kDNA [32, 33], and its full trypanocidal activity is thus dependent on entry into the mitochondrion. Indeed, ISM probably acts preferentially on kDNA rather than nuclear DNA because of its strong accumulation in the mitochondrion [34], a trait it has in common with other di-cationic trypanocides [24,35,36]. This accumulation is driven by the mitochondrial membrane potential ΔΨ_m_ and the strength of this potential reportedly correlates with sensitivity to ISM [37]. Resistance is widely believed to be correlated to differences in drug accumulation in sensitive and resistant strains [38,39], but it is not well understood what causes these differences [3], as at least two transporters are involved (in the plasma membrane and the inner mitochondrial membrane), as well as ΔΨ_m_ and, potentially drug efflux mechanisms. In the current manuscript we show that, in *T*. *brucei*, high levels of ISM resistance can readily be selected for *in vitro* and identify a single point mutation in the γ-subunit of the F_1_-ATPase as a major part of the underlying mechanism; reduced uptake, or increased efflux, played at most a minor role in the level of resistance.

## Methods

### Strains, cultures and growth curves

All parasite cultures used in this study were *Trypanosoma brucei brucei* bloodstream forms of strain Lister 427/MiTat1.2 (wild-type, Tb427WT) or derived thereof by means of adaptation to drugs and/or transfection with specific genes, as indicated. All strains were cultured under standard conditions (37°C, 5% CO_2_) in HMI-9 medium (Invitrogen, Carlsbad, CA) supplemented with 2 mM β-mercaptoethanol (Sigma-Aldrich) and 10% fetal bovine serum (FBS; PAA laboratories, Etobicoke, ON, Canada), as described [40]. ISM was used in the form of Samorin, donated by Merial and the wild-type strain Tb427WT was adapted to high levels of ISM resistance by stepwise increases in the medium concentration of the drug, starting at 0.05 nM, exactly as described [41,42], over several months. Briefly, cell cultures were seeded in 1 ml HMI-9/FBS at a density of 5 × 10^5^ cells/ml in 24-well culture plates and cultured in the presence of ISM for up to 4 days, and then microscopically inspected. If the cells were motile, morphologically normal and had grown at a reasonable rate, the parasites would be reseeded to fresh HMI-9/FBS in 3 wells, containing ISM at the same, double, or half the concentration. If the culture had not grown the cells would be placed in wells containing the same and half the ISM concentration. Following this incremental increase protocol, two strains were generated: ISMR1 was cloned out when the resistance level reached 1 μM, and continuously grown in 1 μM ISM for an additional 14 passages; ISMR15 was cloned out by limiting dilution when the resistance reached 15 μM and also passaged 14 times more in medium containing this concentration of ISM. Both were stored in liquid nitrogen and subsequently grown in normal HMI-9 medium without drug pressure. The level of resistance (and cross-resistance with other trypanocides) was confirmed periodically using the Alamar blue assay (see below) and found to be highly stable for both strains. Ethidium bromide was purchased from Sigma-Aldrich. All cell lines were cloned from single cell by limiting dilution before use in experiments. For the determination of *in vitro* growth curves, cells were seeded at a density of 2 × 10^4^ cells/ml and cell densities were counted every 12 h using a hemocytometer.

### Mitochondrial membrane potential

The mitochondrial membrane potential ΔΨ_m_ was measured by flow cytometry exactly as described [35,43], using the fluorescent indicator dye tetramethylrhodamine ethyl ester (TMRE, Sigma-Aldrich) at 25 nM on a FACSCalibur flow cytometer. Cell cultures were centrifuged and resuspended at 1 × 10^6^ cells/ml in HMI-9/FBS; control samples were also resuspended with 100 nM valinomycin (Sigma-Aldrich). After incubation at 37°C and 5% CO_2_, 1 ml of cells (1 × 10^6^ cells) was centrifuged for 10 min at 4500 rpm (22°C). The pellet was re-suspended in 1 ml of PBS containing 25 nM TMRE and incubated at 37°C for 30 min in the absence of the test compounds. Samples were subsequently placed on ice for at least 30 min before analysis by flow cytometry using the FL2-Height detector and CellQuest software. The detector was calibrated so that the peak of control cells (Tb427WT not exposed to any test compound) was set at 100 arbitrary units, i.e. with 50% of cells at >100 A.U. and 50% at <100 A.U. The data are presented as percent of the cell population with a fluorescence >100 A.U. [24].

### Drug sensitivity assays

Drug sensitivity assays used the dye resazurin sodium salt (Alamar Blue) (Sigma-Aldrich) as described [44] with small changes. Briefly, 96-well plates were set up with a doubling dilution of test compounds in 100 μl HMI9/FBS, leaving the last well drug-free, to which 100 μl of cell suspension were added to give a final cell density of 5 × 10^3^ cells/ml. The plates were incubated at 37°C/5% CO_2_ for 72 h after which 20 μl of resazurin solution (125 μg/ml in phosphate-buffered saline, pH 7.4) was added followed by another incubation for 18 h. Fluorescence was then measured using a FLUOstar Optima plate reader (BMG Labtech, Durham, NC), at excitation and emission wavelengths of 544 and 620 nm, respectively. Data were analyzed using Prism 5.0 (GraphPad) and plotted to a sigmoidal curve with variable slope.

### Uptake and efflux of ISM

Uptake of ISM was measured by incubation of trypanosomes with ISM in a defined assay buffer (AB; 33 mM HEPES, 98 mM NaCl, 4.6 mM KCl, 0.55 mM CaCl_2_, 0.07 mM MgSO_4_, 5.8 mM NaH_2_PO_4_, 0.3 mM MgCl_2_, 23 mM NaHCO_3_ and 14 mM glucose, pH 7.3) followed by separation by centrifugation through an oil layer (7:1 dibutylphthalate/mineral oil (v/v); Sigma-Aldrich), collecting a cell pellet with internalized and/or associated test compound for quantification [45,46]. Fluorescence due to accumulation of ISM was measured using a FLUOstar Optima plate reader (λ_em_ 620 nm; λ_exc_ 355 nm [47]) and quantified using a standard curve of known ISM concentrations, as previously described for fluorescent diamidines and ethidium [48,49]. Briefly, 100 μl of a suspension containing 1 × 10^7^ trypanosomes in assay buffer was carefully added to 100 μl of 20 μM ISM in AB layered over 100 μl of the oil mix in a 1.5-ml microfuge tube. The incubation was allowed to proceed for a predetermined time at room temperature and terminated by centrifugation at 12,500 × g for 1 min. The top layer of assay buffer and the oil layer were carefully removed by capillary suction and the pellet was solubilized in 50 μl of a 0.1 N HCl/methanol (1:8 v/v) mixture (1 h at room temperature). The samples were then transferred to a 96-well plate and the fluorescence determined.

### Fluorescence microscopy

Nuclei and kinetoplasts were visualized by fluorescence microscopy as described [24]. Briefly, trypanosome cultures were adjusted to 5 × 10^5^ cells/ml in HMI-9 and 50 μl was spread out on a microscope slide and allowed to air dry. The slides were placed in ice-cold methanol overnight at -20°C and subsequently dried at room temperature. The preparation was rehydrated for 5 min with 1 ml PBS, which was removed by tipping the slide to its side prior to the addition of 20 μl Vectashield mounting medium containing 1 μg/ml DAPI (4′,6-Diamidino-2-phenylindole dihydrochloride; Vector Laboratories, Burlingame, CA), before covering with a cover slip. The slide was then viewed using a DeltaVision fluorescence microscope.

### Amplification of kinetoplast DNA markers

Full genomic DNA (nuclear and kinetoplast) from *T*. *b*. *brucei* was obtained using the Nucleospin tissue kit (Machery-Nagel, Düren, Germany) according to the manufacturer’s instructions, quantified using a NanoDrop ND1000 spectrophotometer, and stored at -20°C. PCR of kinetoplastid and nuclear markers for the verification of kinetoplast in certain strains used Go Taq polymerase (Promega) using primers and conditions as listed in S1 Table of the Supporting Information.

### Sequencing of the ATPase subunit γ

The full γ-ATP synthase coding sequences, plus 35–90 bp of UTR on either side, were amplified from Tb427WT gDNA, and gDNA from the ISM-resistant strains ISMR1 and ISMR15, using the high-fidelity proofreading polymerase Phusion (Finnzymes; primers given in S1 Table). The amplicons were ligated into the pGEM-T Easy sub-cloning vector (Promega) and eight clones for each strain were sequenced using standard procedures (Source BioSciences, Nottingham, UK). The sequencing data were aligned and compared using CLC Genomics editing software (CLCbio).

### Expression of wild-type and S284* ATPase subunit γ in *T*. *b*. *brucei*

F_1_F_o_-ATPase subunit γ (systematic gene ID Tb927.10.180, www.tritrypdb.org) S284* mutants were generated in the Tb427WT cell line using gene replacement constructs (Matt Gould and Achim Schnaufer, manuscript in preparation). Briefly, a single round of transfections was carried out to replace one wild type γ allele with a S284* mutant allele. In parallel, an otherwise isogenic control cell line was generated but without the S284* mutation.

### Infectivity in mice

Cohorts of 5 female ICR mice (Harlan, UK) per cell line were inoculated intraperitoneally with 200 μl PBS containing 2 × 10^5^ trypanosomes in order to test whether the adapted strains would be capable of establishing an infection. Parasitaemia was monitored in single drops of blood drawn from a tail puncture. Mice were humanely euthanized by CO_2_ inhalation when judged to have reached a terminal parasitaemia; the experiment was ended after taking the blood sample on day 5 post-infection and all mice euthanized with CO_2_.

### Ethics statement

The single experiment to test infectivity of the ISM-resistant strains was performed at the University of Glasgow Joint Research Facility under the supervision of trained professionals; the facility is regularly inspected by a UK Home Office Inspector and adheres to all national and international regulations as stipulated by the UK Home Office for animal care and in accordance with the Animals (Scientific Procedures) Act 1986 as amended in 2012. The procedure had been expressly approved and licensed by the UK Home Office (project license PPL 60/3760 ‘Biochemistry, genetics and immunology of parasitic protozoa’) and the experiment was performed by a trained animal technician under his personal Home Office license (PIL601/12386). Since the aim of the experiment was only to establish whether the new trypanosome lines were infective to mice, the duration of the experiment was kept to the minimum (5 days) needed to establish this, after which the mice were humanely euthanized by CO_2_ inhalation; any mouse found to have high parasitaemia during the experiment, or show any signs of suffering was similarly euthanized, as is standard procedure.

## Results

### Generation and characterization of isometamidium resistant bloodstream form *T*. *b*. *brucei*

**Fig 1:**
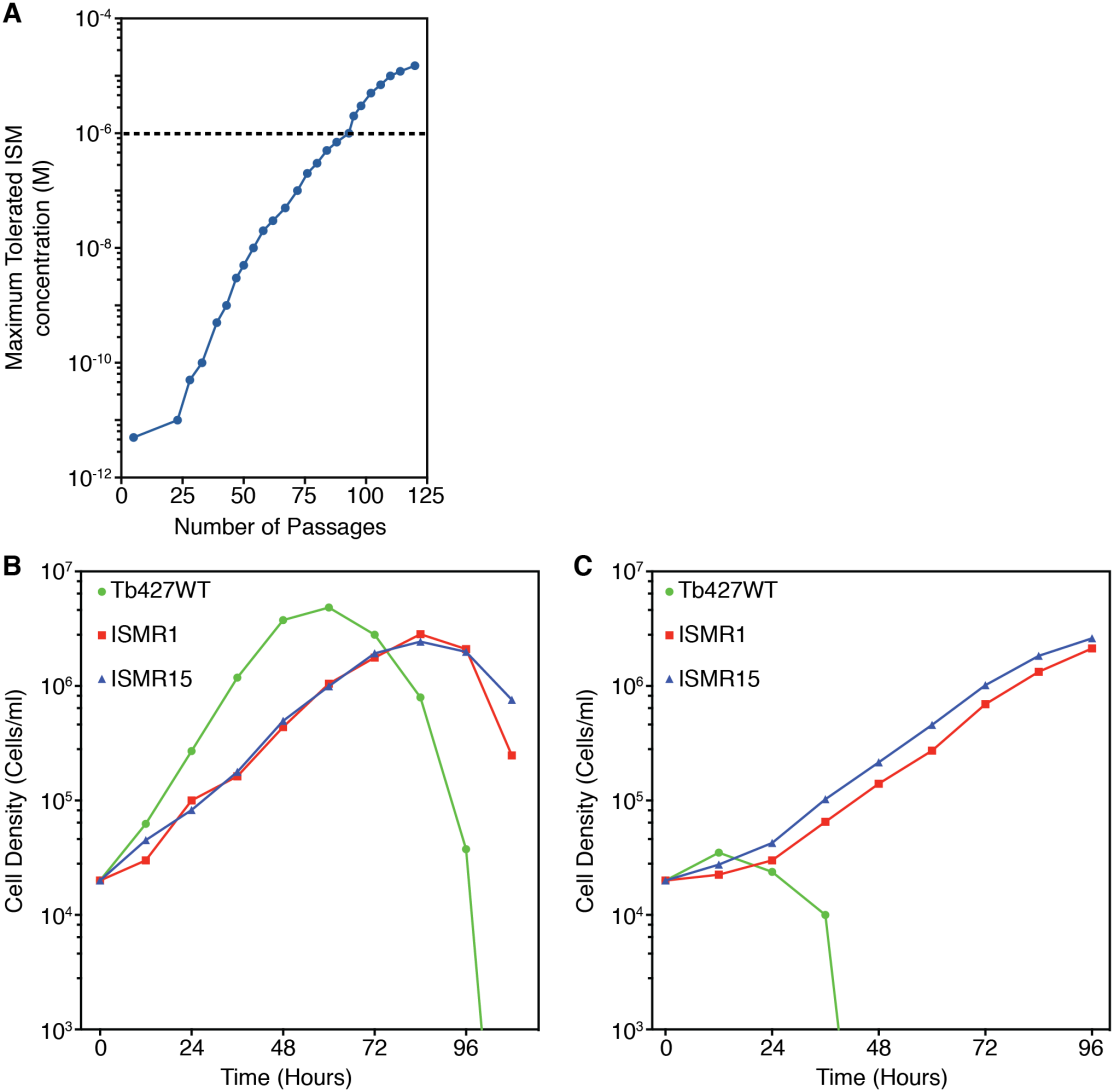
Induction of resistance to ISM. (A) Wild type *T*. *b*. *brucei* strain 427 bloodstream form cells (Tb427WT) were continuously cultured in gradually increasing concentrations of ISM. The maximum tolerated ISM dose was determined periodically. The dashed line represents the point that cell line ISMR1 was cloned by limiting dilution. (Panels B and C) Growth of ISM adapted cell lines in the absence (B) or presence (C) of 5 μM ISM. Values are the average of two independent determinations in parallel cultures.

Resistance to ISM was induced in bloodstream form *T*. *b*. *brucei* Lister 427 strain (Tb427WT) by culturing with incrementally increasing, sub-lethal concentrations of the drug in standard *in vitro* medium. Drug exposure started with an ISM concentration of 50 pM, a concentration that reduced the growth rate but did not kill the trypanosomes. After 9 months of continual culturing and 93 passages, the cells had adapted to tolerate 1 μM ISM (Fig 1A); a clonal population was then obtained by limiting dilution and designated ISMR1. After a further 3 months culturing and 19 passages in ISM, a second clone was obtained that could tolerate 15 μM and was designated ISMR15. The routine maintenance of the clonal cell lines was then carried out in the absence of ISM drug pressure.

Under standard culturing conditions and in the absence of ISM pressure, ISMR1 and ISMR15 grew at the same rate, but more slowly than the parental strain Tb427WT, with ISMR1 and ISMR15 reaching the stationary phase of growth around 24 hours after Tb427WT (Fig 1B). In the presence of 5 μM ISM the Tb427WT cells died after 48 hours; however, ISMR1 and ISMR15 continued to grow (Fig 1C), apparently unaffected by a normally lethal concentration of ISM and demonstrating that the adaptation(s) giving rise to ISM resistance were stable and retained even after culturing without ISM pressure.

An *in vitro* drug sensitivity assay established that ISMR1 and ISMR15 were, respectively, 7270- and 16,060-fold resistant to ISM when the EC_50_ values of the resistant clones were compared to those of the parental Tb427WT strain (Table 1). Major cross-resistance was also displayed by ISMR1 and ISMR15 to ethidium bromide, a member of the same phenanthridine class of compounds as ISM, with 829-and 519-fold increases in EC_50_ values, respectively, as well as to the diamidine compound diminazene (38- and 43-fold resistant, respectively). Minor loss of sensitivity was shown for pentamidine for ISMR1 (2.5-fold), but not for ISMR15.

**Table 1:** Resistance profile of ISM-adapted cell lines and the parental cell line Tb427WT. EC_50_ values are given as mean of at least 5 independent determinations and SEM. The resistance factor (RF) is the ratio of the EC_50_ values of the adapted strain and the wild-type control. Statistical significance was determined using a two-tailed unpaired t-test.

	Tb427WT	ISMR1		ISMR15	
Compound	Mean EC_50_ (nM)	Mean EC_50_ (nM)	Resistance Factor *vs*. Tb427WT	Mean EC_50_ (nM)	Resistance Factor *vs*. Tb427WT
Isometamidium	0.14 ± 0.04	1020 ± 100	7270***	2260 ± 250	16,060***
Ethidium Bromide	1.3 ± 0.1	1120 ± 43	829***	700 ± 30	519***
Diminazene	20.5 ±1.1	770 ± 140	38.1***	875 ± 43	42.7***
Pentamidine	2.5 ± 0.2	6.3 ±0.9	2.5***	2.1 ± 0.5	0.84
Oligomycin	250 ± 14	1080 ± 60	4.3***	1100 ± 70	4.4***
Valinomycin	0.59 ±0.15	0.27 ±0.07	0.46	0.26 ± 0.05	0.45

Asterisks represent P-values for statistically significant resistance, as calculated using a one-tailed unpaired Student’s t-test:

***, P-value <0.001.

### ISMR1 and ISMR15 have reduced mitochondrial membrane potentials and ISM uptake

**Fig 2:**
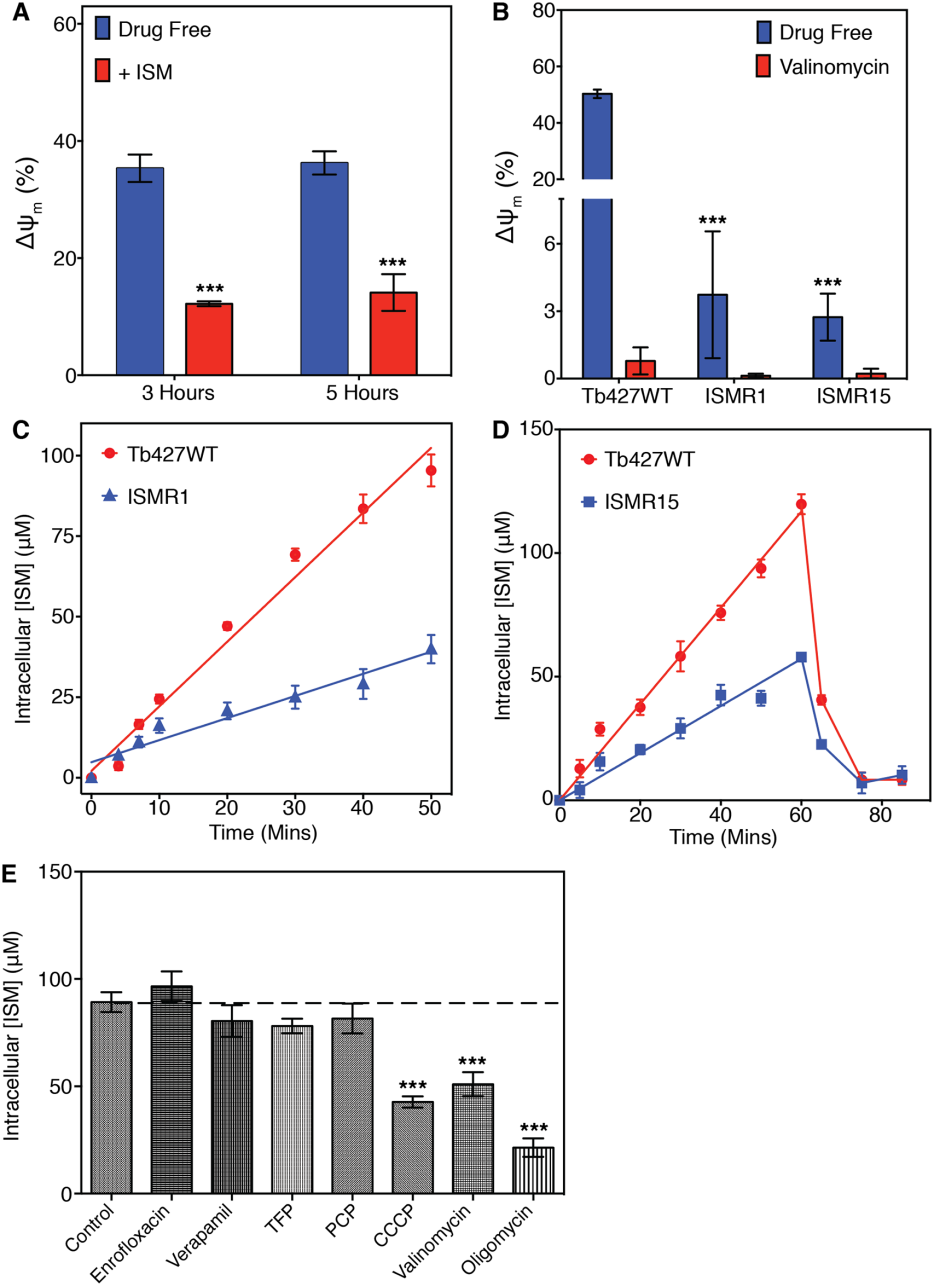
Adaptation giving resistance to ISM reduces the mitochondrial membrane potential and ISM uptake capacity. (A) Mean mitochondrial membrane potential (ΔΨm) with SEM of Tb427WT trypanosomes incubated with or without 0.5 μM ISM for 3 or 5 hours. ΔΨm is recorded in arbitrary units (A.U.) of fluorescence, and flow cytometer is calibrated at the start of the experiment to have exactly 50% of cells with a value of 100 A.U. and over for the drug-free cells (control at t = 0 h). ***, P-value <0.001 (Student’s t-test, n≥3) of the ISM-treated cells relative to their untreated controls. (B) Mean ΔΨm with SEM of ISM resistant cell lines compared to the parental Tb427WT. Valinomycin was used as a positive control. ***, P-value <0.001 (Student’s t-test, n≥3) relative to WT control. (C) Graph showing the means (and SEM) of 3 independent experiments measuring uptake of ISM by the parental Tb427WT and ISMR1 cell lines. For each of the experiments single cell cultures of either Tb427WT or ISMR1 were incubated in 10 μM ISM, which were sampled in triplicate at the indicated times for the determination of intracellular ISM. (D) Graph showing the means (and SEM) of 3 independent experiments showing uptake and efflux of ISM by the Tb427WT and resistant ISMR15 strains. After incubation with 10 μM ISM for 60 minutes, the cells were centrifuged, washed and resuspended in medium without drug. The intracellular concentration of ISM was monitored in triplicate periodically before and after the removal of drug from the medium. (E) Inhibition of ISM uptake in the presence of potential inhibitors. Tb427WT trypanosomes were incubated for 20 minutes with 10 μM ISM in the absence (Control) or presence of 50 μM enrofloxacin, verapamil, TFP, PCP, CCCP, oligomycin or 1 μM valinomycin. Data presented are mean intracellular ISM concentration with SEM; ***, P-value <0.001 (Student’s t-test, n≥3) relative to untreated controls.

In order to investigate whether ISM affects the mitochondrial membrane potential (ΔΨ_m_), the uptake of the fluorescent compound TMRE was observed using flow cytometry. TMRE is a cell-permeable orange dye that is positively charged and accumulates in functional mitochondria due to their polarized state; if the ΔΨ_m_ falls or is reduced, less TMRE accumulates and fluorescence is lowered [35]. Incubation of Tb427WT cells with 0.5 μM ISM for 3 or 5 hours resulted in significantly lowered TMRE fluorescence compared to non-ISM exposed cells (Fig 2A), suggesting that ISM disrupted the ΔΨ_m_ of trypanosomes. In non-ISM exposed ISMR1 and ISMR15 cells, the fluorescence due to TMRE accumulation was also significantly reduced compared to wild type, showing that the basal steady-state ΔΨ_m_ was much lower in the ISM resistant cells (Fig 2B).

As previous studies have demonstrated that ISM accumulates in the mitochondrion of trypanosomes [34,38], the lowered ΔΨ_m_ of the resistant cells may have reduced the uptake of the dicationic ISM into this organelle; consequently, the uptake of ISM was monitored through the intrinsic fluorescent properties of the drug. Uptake of ISM appeared to be linear for at least 50 minutes, accumulating to far higher intracellular concentrations than the extracellular concentration being applied (Fig 2C). This implies an accumulative mechanism for ISM uptake through either active transport or segregation in the cell. In several experiments that monitored ISM uptake over 50 minutes in Tb427WT and ISMR1, the average rate of ISM accumulation in ISMR1 was consistently 2.5–3.5-fold lower than the control, but still linear for the duration of the experiment and accumulating against the concentration gradient (Fig 2C). For the ISMR15 cell line, with an even higher level of ISM resistance, very similar rates of ISM accumulation were observed (Fig 2D, 0–60 mins time points).

Despite linearity of the uptake, these measurements clearly represent a consistent ongoing net accumulation of ISM, being the sum of uptake minus any cellular efflux, rather than simply initial rates of transport. In order to investigate whether efflux was a moderating factor to the net uptake, and whether it could help explain the resistance phenotype, cells from Tb427WT and from the most resistant strain, ISMR15, were loaded by incubation with 10 μM ISM for 60 minutes (again displaying linear increase to above the extracellular concentration), after which the cells were washed into fresh medium and cellular ISM content monitored. Both strains rapidly lost most of the ISM content over a similar period of time due to efflux, despite having differing final intracellular ISM concentrations at the washing step (Fig 2D); consequently, it is unlikely that a difference in efflux mechanism constitutes the main, or a significant portion of, the ISM resistance mechanism.

In addition, the ISM efflux transporter was not sensitive to the ABC transporter inhibitors verapamil, prochlorperazine (PCP) and trifluoperazine (TFP), as their application at 50 μM did not affect net ISM accumulation levels. Nor did the antibiotic enrofloxacin, previously reported to sensitize African trypanosomes to ISM [10], change the intracellular ISM concentration (Fig 2E). In contrast, three agents that reduce the mitochondrial potential, oligomycin, CCCP and valinomycin, all significantly inhibited ISM accumulation in Tb427WT cells (Fig 2E), consistent with findings reported for *T*. *congolense* [37]. Interestingly, in the *in vitro* drug sensitivity assay, significant resistance was displayed by ISMR1 and ISMR15 cells against oligomycin (4.0- and 4.1-fold resistant respectively; Table 1), a compound that specifically targets the F_1_F_o_-ATPase in mitochondria [50], suggesting a role for this protein complex in the ISM resistance adaptations.

### ISMR1 and ISMR15 have lost their kinetoplast DNA

**Fig 3:**
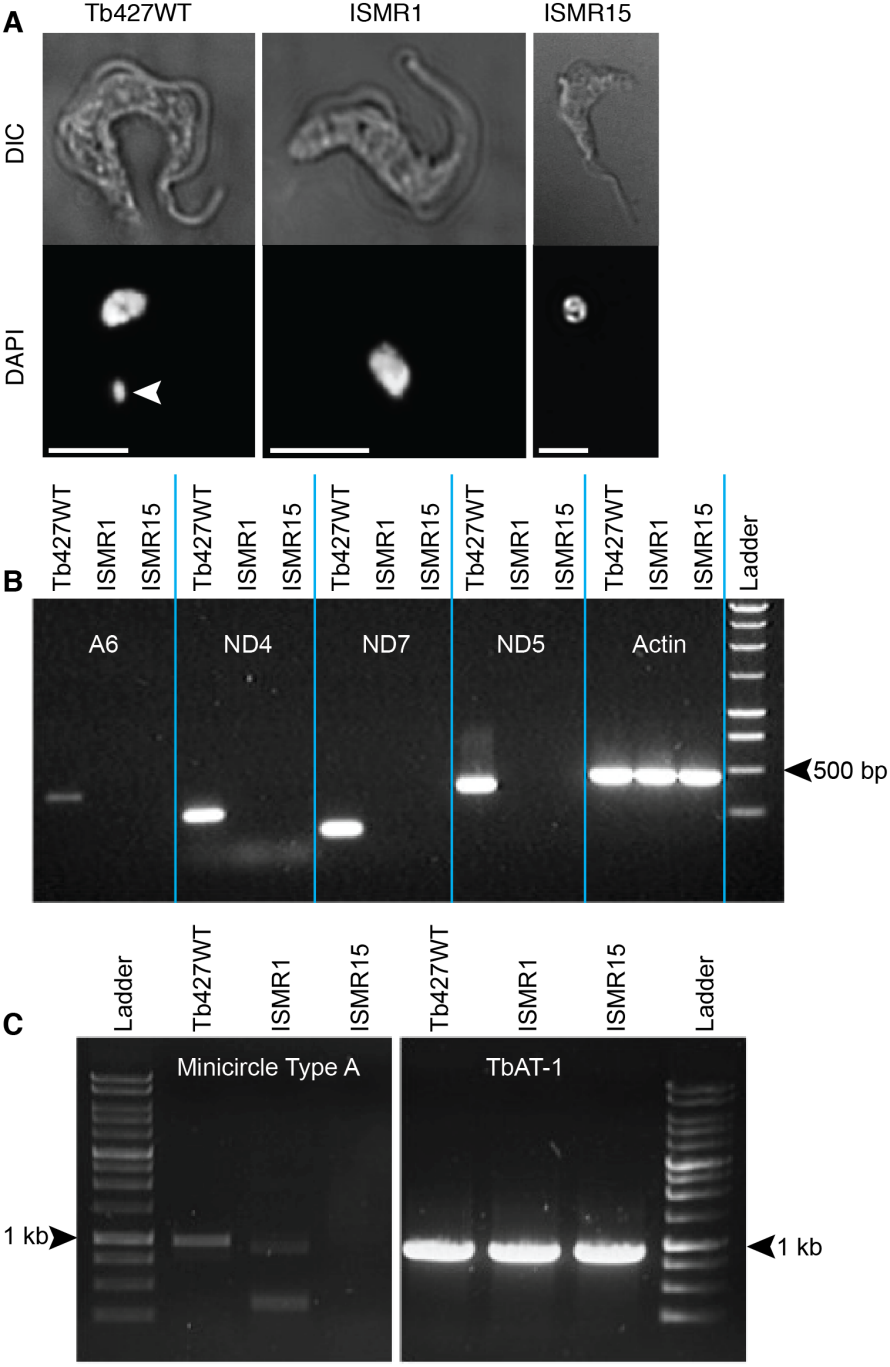
ISM resistant cell lines have lost their kinetoplasts. (A) Fluorescence microscopy of DAPI-stained Tb427WT and ISMR1 and ISMR15 cell lines. The white arrowhead indicates the kinetoplast, which is absent in ISMR1 and ISMR15. The white scale bar represents a length of 5 μm. (B) Electrophoresis gel of PCR products of kinetoplast-encoded genes and nuclearly encoded actin. Genomic DNA was extracted from the parental Tb427WT strain as well as from the ISM resistant ISMR1 and ISMR15 strains and subjected to PCR amplification using primers specific for the gene fragments stated (S1 Table). (C) Like frame B but using primers specific for minicircles, using the TbAT1 gene as a positive control for a nuclearly-encoded single copy gene [62].

In trypanosomes the mitochondrial genome is a highly organized structure within the mitochondrion, called the kinetoplast, which can be easily visualized by staining with the DNA-binding fluorophore DAPI. Fluorescence microscopy of fixed cells after staining clearly showed the presence of the kinetoplast and nucleus in wild type trypanosomes; in contrast, for ISMR1 and ISMR15, no kinetoplasts were observed in any cell (Fig 3A and S1 Fig).

In order to determine whether the mitochondrial genome of ISMR1 and ISMR15 had been completely eliminated or merely disaggregated and dispersed throughout the mitochondrion or cytosol, rendering it undetectable by DAPI staining, DNA was extracted and subjected to PCR analysis. Fragments of 4 genes known to be encoded only by the kinetoplast maxicircles were specifically amplified from parental Tb427WT DNA (Fig 3B). However, none of the 4 kinetoplast encoded gene fragments could be amplified from ISMR1 or ISMR15 DNA, whereas the nuclearly encoded actin gene was readily amplified from all 3 strains tested. Similarly, PCR-based detection of representative type-A-like minicircles confirms their absence in ISMR15 (Fig 3C). Two fainter products of shorter size in ISMR1 likely represent non-specific bands, or, less likely, type-A-like minicircles that have suffered internal deletions. Either possibility is consistent with completed or ongoing loss of kDNA in the ISM resistant strains. This demonstrated that there was no functional mitochondrial genome in either ISMR1 or ISMR15, as this requires both intact maxicircles and minicircles. In the case of ISMR15 kinetoplast DNA had been entirely lost from the cell, whereas in ISMR1 it cannot be ruled out that some remnants of minicircles, but not of maxicircles, may have remained.

### Identification in ISMR1 and ISMR15 and characterisation of a mutation in the ATP synthase γ subunit of the F_1_F_o_ -ATPase complex

It has been demonstrated that the kinetoplast is normally essential in bloodstream form trypanosomes, but also that the loss of the kinetoplast can be compensated for by certain mutations in the carboxyl terminal part of the nuclearly encoded γ subunit of the F_1_F_o_-ATPase complex [51]. Such mutations result in substantial loss of ISM sensitivity [33]. To assess whether similar mutations have been selected for in the present study, the ATPase subunit γ gene was amplified from ISMR1, ISMR15 and the parental Tb427WT strain using a proofreading polymerase, cloned and sequence. Two mutations were identified, with the first being a substitution at base pair 37 of the open reading frame, of only one of the two alleles (S2A Fig), resulting in an amino acid change from glutamic acid to lysine. The second mutation was homozygous and found at base pair 851 with a cytosine substituted for an adenine (S2B Fig). This point mutation resulted in the generation of a stop codon, terminating the peptide sequence prematurely at amino acid position 284 (S284*), truncating subunit γ by 22 residues at the carboxyl terminal end. The identified alleles were assigned the following GenBank accession numbers: F_1_F_o_-ATPase subunit γ G37A/S284* double mutant (KX444504); S284* single mutant (KX444505); Tb427WT (KX444506).

**Fig 4:**
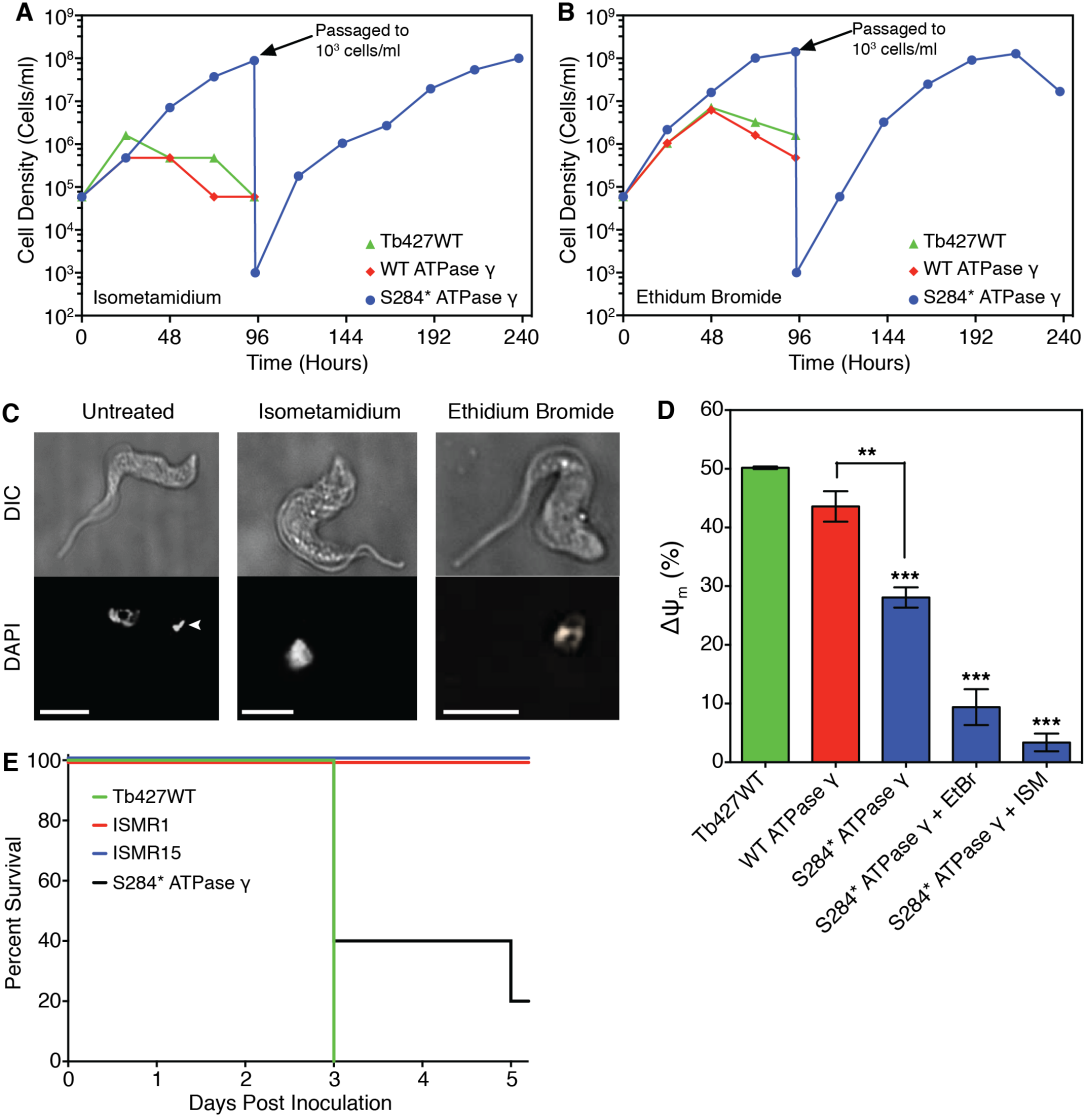
Characterisation of S284* mutation in ATP synthase subunit γ identified in ISM resistant ISMR1 and ISMR15 trypanosomes. Tb427WT trypanosomes were genetically manipulated to replace one allele of subunit γ with either a version that contained the S284* mutation or another wild type copy as a control, and then cultured in the normally lethal concentration of either 20 nM ISM or ethidium bromide (Panels A-D). (Panels A and B) Growth of the genetically modified trypanosome strains in the presence of ISM (A) or ethidium bromide (B) alongside the parental Tb427WT cell line. (C) Fluorescence microscopy of DAPI-stained S284* subunit γ expressing trypanosomes before and after 7 days exposure to ISM or ethidium bromide. The white arrowhead indicates the kinetoplast (absent in the drug-treated cells); the white scale bar represents 5 μm. (D) The mitochondrial membrane potential (ΔΨm) of S284* subunit γ expressing trypanosomes, treated and untreated with ISM or ethidium bromide (EtBr) for 7 days compared to an otherwise isogenic strain expressing non-mutated subunit γ and the parental Tb427WT cell line. Data presented is the mean with SEM; **, P-values <0.01; ***, P-values <0.001 (Student’s t-test, n≥3), relative to Tb427WT unless otherwise indicated. (E) The *in vivo* virulence of the ISM resistant ISMR1 and ISMR15 cell lines as well as the S284* subunit γ expressing strain with the parental Tb427WT as a control. Five mice were inoculated intraperitoneally for each trypanosome strain and monitored daily for parasitaemia, with those mice reaching terminal parasitaemia euthanized. Data presented shows percentage of surviving mice in each cohort.

To test whether the S284* mutation identified in ISMR1 and ISMR15 might be involved in ISM resistance, two otherwise isogenic cell lines were generated in which one subunit γ allele was replaced with either a S284*-mutated version or a wild type copy as a control. The S284* and wild type subunit γ replacement cell lines, along with the unmodified parental Tb427WT strain, were cultured in the presence of either 20 nM ISM or 20 nM ethidium bromide—concentrations that are normally lethal to bloodstream form trypanosomes. After 2 days of culturing, the Tb427WT strain and the wild type ATPase-γ replacement cell lines stopped growing and began to die; in contrast, the S284* mutant-expressing cell line appeared to be completely unaffected by ISM or ethidium bromide and maintained continuous growth (Fig 4A and 4B).

After 7 days of growth in ISM or ethidium bromide, the surviving S284* subunit γ cell lines were fixed, stained with DAPI and assessed by fluorescence microscopy for the presence of kinetoplasts. Neither the ISM nor the ethidium bromide exposed cell lines had retained a visible kinetoplast, whereas untreated cells of the same strain still had a clearly identifiable kinetoplast (Fig 4C). Similarly, kinetoplast encoded genes were no longer detectable after exposure to ISM or ethidium bromide (S3 Fig), demonstrating that expression of the mutated subunit γ in itself did not induce loss of the kinetoplast, but that it was sufficient to compensate for its loss when induced by drug pressure.

The ΔΨ_m_ of the S284* ATPase-γ trypanosomes was significantly lower than for the parental wild type and wild type ATPase-γ replacement cell lines; this reduction was further amplified on the loss of the kinetoplast after ISM or ethidium bromide exposure (Fig 4D).

*In vitro* drug sensitivity assays determined that expression of the S284* mutated subunit γ resulted in a similar drug resistance profile as ISMR1 and ISMR15. Significant resistance was displayed to the phenanthridine compounds ISM and ethidium bromide (104- and 374-fold, respectively, compared to the parental Tb427WT; Table 2). Lower levels of resistance were determined for the diamidine compounds diminazene (5.3-fold) and pentamidine (2.6-fold) and the F_1_F_o_-ATPase inhibitor oligomycin (5.2-fold)). However, for ISM, ethidium bromide and diminazene, the EC_50_ values were still significantly lower than for the ISMR1 and ISMR15 cell lines (all P<0.0001, n = 5–11; compare EC_50_ values in Table 1 with those in Table 2).

**Table 2:** Drug sensitivity profile of Tb427WT cells expressing either the wild-type F_1_ subunit γ gene, or the same gene with mutation S284*, either exposed to ethidium bromide or ISM, or not exposed to any trypanocide. All data are the mean of three independent experiments and SEM.

	WT ATPase γ	S284* ATPase γ Unexposed		S284* ATPase γ Isometamidium Exposed			S284* ATPase γ Ethidium Bromide Exposed		
Compound	Mean EC_50_ (nM)	Mean EC_50_ (nM)	Resistance Factor *vs*. WT ATPase γ	Mean EC_50_ (nM)	Resistance Factor *vs*. WT ATPase γ	Resistance Factor *vs*. Unexposed	Mean EC_50_ (nM)	Resistance Factor *vs*. WT ATPase γ	Resistance Factor *vs*. Unexposed
Isometamidium	0.31 ± 0.06	31.9 ± 6.1	104*	172 ± 59	560***	5.4***	162 ± 46	529***	5.1***
Ethidium Bromide	1.49 ± 0.21	554 ± 50	372***	361 ± 16	242***	0.65	322 ± 36	216***	0.58
Diminazene	20.3 ± 0.8	108 ± 6.4	5.3**	132 ± 24	6.5***	1.2	136 ± 26	6.7***	1.3
Pentamidine	2.8 ± 0.3	7.22 ± 0.66	2.6**	2.3 ± 0.3	0.82	0.32	2.38 ± 0.73	0.84	0.33
Oligomycin	270 ± 33	1410 ± 90	5.2***	872 ± 282	3.2**	0.62	1180 ± 170	4.4***	0.83

Asterisks represent P-values for statistically significant resistance, as calculated using a one-tailed Student’s t-test:

*, P-value <0.05;

**, P-value <0.01;

***, P-value <0.001.

The loss of the kinetoplast by exposure to ethidium bromide or ISM resulted in a further significant loss of sensitivity to ISM (P<0.001), while still remaining significantly less resistant than ISMR1 (P<0.001), whose high level of resistance must be multifactorial, consistent with the gradually increasing tolerance during the induction of the resistance (Fig 1A). In contrast, the loss of the kinetoplast rendered the S284* subunit γ cells somewhat more sensitive to ethidium bromide (~1.6-fold; P<0.02) and pentamidine (~3-fold, P<0.001), compared to the non-drug exposed cells (Table 2).

### ISMR1, ISMR15 cell lines and the S284* ATPase-γ expressing trypanosomes remain viable in vivo

5 mice per group were inoculated intraperitoneally with 2 x 10^5^ parental Tb427WT, ISMR1, ISMR15 or S284* subunit γ expressing trypanosomes, respectively, and monitored daily for parasitaemia. After 3 days all of the mice inoculated with the parental wild type cells had reached a terminal parasitaemia (Fig 4E), while 3 out of the 5 mice infected with the S284* subunit γ expressing cell line had reached the same stage in the same time. A fourth mouse infected with the S284* subunit γ cell line had reached a terminal parasitaemia by the fifth day. In contrast, while trypanosomes were detected in the blood of all mice inoculated with ISMR1 or ISMR15 by the second day, the parasitaemia remained at a relatively constant, but non-lethal level for the remainder of the experiment. The difference in parasitaemia between the ISM-adapted cell line and the S284* mutant are most likely attributable to the fact that in the in vitro adapted cell lines multiple mutations must have occurred, leading to a higher level of resistance than observed for the genetically modified S284* line. No attempt was made to observe parasitaemia beyond 5 days, after which the surviving mice were euthanized.

## Discussion

Investigations into adaptations allowing bloodstream form trypanosomes such as *T*. *evansi* and *T*. *equiperdum* to survive without mitochondrial DNA (the kinetoplast) identified enabling mutations in the γ subunit of the F_1_F_o_ -ATPase complex [21,51–53]. These mutations completely compensated for the loss of the normally essential kinetoplast-encoded ATPase subunit A6 by uncoupling, at least functionally if not physically, the now incomplete proton-pumping moiety (F_o_) from the ATP hydrolyzing part (F_1_) of the ATPase complex [51]. Not only did these mutations result in independence from the kinetoplast, it was also demonstrated that expression of a mutated subunit γ, in this case through genetic manipulation, resulted in significant resistance to ISM and other phenanthridines as well as to some diamidine compounds [33]. It was hypothesized that these mutations were responsible for the observed innate resistance to ISM displayed by some animal-infective strains of trypanosomes and could have relevance for other treatment failures in the field. The current study aimed to investigate, in an unbiased approach, whether this and/or other modes of resistance could be acquired by *T*. *b*. *brucei* without genetic manipulation, by stepwise selection through exposure to gradually increasing concentrations of ISM.

Significant resistance to ISM was readily generated, albeit over a period of several months. However, as ISM has a reported half-life in the blood of cattle of over 3 weeks [54], exposure of trypanosomes to sub-lethal concentrations of the drug over a prolonged period of time is a physiological possibility in prophylactically treated animals, especially if ‘top-up’ doses are not given. In addition, counterfeit or sub-standard formulations as well as miscalculation of the effective dosage can all allow ineffective doses of ISM to be administered [55], again exposing trypanosomes to potentially sub-lethal concentrations of the drug over extended periods of time due to its long half-life. We found that high levels of ISM resistance were readily induced *in vitro*. The resultant cell lines ISMR1 and ISMR15 were able to establish and maintain infections *in vivo*. This is consistent with stable (and thus transmitted) ISM resistance reported from the field [7,12]. Nevertheless, it is doubtful that akinetoplastic *T*. *brucei* could complete the life cycle in the tsetse fly and any transmission is likely to be mechanical by biting insects.

Beyond their extreme resistance to ISM, the most striking phenotype of the ISMR1 and ISMR15 cell lines is their complete lack of kinetoplast DNA. The identification of a novel truncation mutation at the C-terminal end of the ATPase subunit γ in the resistant clones is in good agreement with previous work demonstrating that most naturally occurring dyskinetoplastic or akinetoplastic trypanosome strains sequenced to date have modifications in the same protein [51,53]. Further characterisation of the S284* mutation demonstrated that, like the other substitution mutations, expression of this mutation on its own was sufficient to allow bloodstream form trypanosomes to survive without a kinetoplast. Although the introduction of this mutant allele did not automatically lead to loss of the kinetoplast, it enabled the extremely rapid loss of this organelle upon exposure to phenanthridine trypanocides *in vitro*.

The S284* mutation, examined in isolation, also conferred >100-fold resistance to ISM, as well as >350-fold resistance to ethidium bromide, ~5-fold resistance to diminazene and ~2.5-fold resistance to pentamidine; evidence that the single S284* mutation established a significant level of cross-resistance to diamidine class compounds in addition to that shown to the phenanthridines. While considerable resistance levels were recorded with the S284* mutation, the degree of resistance, for ISM especially, was much less than that shown by ISMR1 and ISMR15, suggesting that the mutation in ATPase subunit γ is one of a minimum of two adaptations in these highly ISM-resistant cell lines. It could be argued that if both alleles in the S284* expressing cell line had been mutated then the resistance levels may have been much more dramatic, especially since the *in vitro* selection regime had resulted in such homozygous genotypes. However, an L262P mutation in the same gene resulted in very similar levels of resistance [33], despite in that instance both endogenous subunit γ alleles having been knocked out and the mutated version being ectopically expressed from the tubulin locus. This suggests that gene dosage or expression level ratio of mutant to wild type ATPase subunit γ is probably not a key determinant of the degree of resistance and that a homozygous S284* mutant may confer a limited degree of further resistance. As the fold resistances seen to pentamidine in the ISMR1 and S284* expressing cell lines are approximately the same, it appears that independence from the kinetoplast is likely to be the sole mode of resistance to this drug in the ISMR cell lines and any secondary mechanisms do not affect pentamidine sensitivity.

Another drug resistance mechanism commonly found in African trypanosomes is to limit the accumulation of drug within the cell by adjusting transport of the compound [14,15,56,57], either by reducing uptake or increasing efflux out of the cell. When compared to parental wild type trypanosomes, net uptake of ISM by the ISMR cell lines was significantly lowered. The reduction in ISM accumulation does not appear to be due to an increase in efflux of the compound, however, since after washing out ISM from the trypanosomes’ culture medium the rate by which the intracellular ISM concentration diminished was roughly the same between resistant and sensitive cell lines. Also, the application of inhibitors of ABC transporters (increased expression of which is implicated in multi-drug resistance in many different systems including the related kinetoplastid *Leishmania* [58]) did not enhance accumulation of ISM as one would expect if they were responsible for ISM efflux. However, only a limited panel of ABC transporter inhibitors was tested, as active extrusion was clearly not the main resistance mechanism. Importantly, we found that the antibiotic enrofloxacin, previously reported to chemosensitize *T*. *congolense* to ISM (an effect that was tentatively ascribed to possible competitive inhibition of efflux transporters) [10], had no effect on ISM accumulation in *T*. *brucei*. The ISM-resistance mechanisms of *T*. *brucei* and *T*. *congolense* could of course be different, as reported for diminazene [19] and it is now important that the findings reported here are tested in other animal-infective trypanosomes, particularly *T*. *congolense* and *T*. *vivax*—species much less amenable to *in vitro* culture and genetic manipulation.

As yet, the transporter(s) directly responsible for accumulation and concentration of ISM in trypanosomes has/have not been genetically identified and characterized. Consequently, we cannot rule out that a mutation in, or change in expression of, a putative ISM transporter is responsible for, or contributes to, the overall reduced uptake of the drug into the cell. The ISMR cell lines here described could play an important role in the identification of the ISM transporter(s).

Compounds that did decrease ISM uptake, however, were those that reduce the mitochondrial membrane potential, suggesting that the ΔΨ_m_ is important for the uptake of the drug. This is completely consistent with previous investigations where the transport rate of ISM in *T*. *congolense* was correlated with ΔΨ_m_ in the various strains, which also correlated with sensitivity to the drug [37]. One of the ISM uptake-decreasing compounds used was oligomycin, which acts by inhibiting the proton pumping part of the F_1_F_o_-ATPase [59]. Both ISMR1 and ISMR15 were significantly resistant to this compound, as was the S284* expressing cell line, and this is further confirmation that the mutations in ATPase subunit γ confer independence from the kinetoplast and uncouple the proton pumping part from the ATP hydrolyzing moiety [51].

We also demonstrated that ISM itself reduced the ΔΨ_m_ of wild type trypanosomes. This is not unexpected as it has been shown that ISM accumulates in the mitochondrion [47] and binds to kDNA [34], thereby probably inhibiting the expression of the kinetoplast-encoded A6 subunit of the F_1_F_o_ -ATPase complex. In addition, the uptake of large amounts of ISM, a di-cation, into the mitochondrion, is likely to directly affect ΔΨm.

An additional explanation for the lowered basal ΔΨ_m_ could be that the as yet unidentified secondary ISM resistance mechanism impacts on the ΔΨ_m_, possibly in a way that is unrelated to the F_1_F_o_-ATPase complex. A recent investigation using RIT-seq methodology identified 30 potential candidate genes capable of conferring a certain level of resistance to ISM [60]. The majority of these were components of one of three complexes: V-type H^+^ ATPase (V-ATPase); endoplasmic-reticulum membrane complex (EMC) and Adaptin-3. In trypanosomes, V-ATPase complexes are localized to the lysosome and acidocalcisomes [61]. Surprisingly, mild RNAi-mediated depletion of subunits of the V-ATPase, Adaptin-3 or EMC resulted in cells that were ISM resistant and independent of their kinetoplasts [60]. Furthermore, depletion of V-ATPase subunits desensitized trypanosomes to oligomycin, suggesting some level of redundancy or regulation between the non-mitochondrial V-ATPase and the mitochondrial F_1_F_o_-ATPase. While this study found no measurable change to ISM uptake or ΔΨ_m_ on RNAi of the V-ATPase subunits, it must be noted that the proteins were only partially depleted as full RNAi induction was lethal. It is possible that different adaptations to any of the V-ATPase subunits, beyond expression level, could have a more obvious effect on ΔΨ_m_ while still retaining cell viability and may form part or all of the secondary ISM resistance mechanism identified in ISMR1 and ISMR15.

In conclusion, we have validated dominant-negative mutations to ATPase subunit γ as having the potential to spontaneously arise in the field in response to exposure to sub-lethal concentrations of ISM, conferring a high level of resistance to phenanthridine drugs and significant cross-resistance to diamidine trypanocides. In addition, other mechanism(s) of resistance can also occur, which impact on ΔΨ_m_ and on the uptake of ISM. Further studies will investigate whether mutations in ATPase-γ are involved in ISM resistance in the field.

## Supporting Information

S1 Fig Micrograph showing multiple cells of either ISMR1 or ISMR15 in various cell cycle stages. None of the cells in the population contain a kinetoplast.
(TIF)

S2 Fig Sequence alignments showing mutations in ATP synthase subunit γ identified in the ISM resistant ISMR1 and ISMR15 strains compared to the parental Tb427WT strain. The full length ORF of subunit γ was amplified using a proofreading polymerase. The products were A-tailed using Taq polymerase and cloned into the pGEM-T Easy vector. Eight clones from each trypanosome cell line were sequenced and aligned; the sequence for strain 427 was taken from the TriTryp genome database (www.tritrypdb.org) and used as a reference sequence for all alignments. (A) Sequence of nucleotides 1–60. (B) Sequence of nucleotides 820–880.
(TIF)

S3 Fig Electrophoresis gel of PCR products of kinetoplast-encoded genes and nuclearly-encoded actin. Genomic DNA was extracted from Tb427WT trypanosomes with one allele of ATP synthase subunit γ endogenously replaced with a version containing the S284* mutation, as well as from the same strain after 7 days exposure to either 20 nM ISM or ethidium bromide. PCR amplification was carried out using primers specific for the genes stated (S1 Table).
(TIF)

S1 Table Oligonucleotide primer sequences used in this study. (DOCX)

## References

[1] Swallow B. Impacts of trypanosomiasis on African agriculture. PAAT Technical and Scientific Series; 2000. ISBN 92-5-104413-9. http://www.fao.org/ag/againfo/programmes/en/paat/documents/papers/Paper_1999.pdf

[2] La Greca F, Magez S. Vaccination against trypanosomiasis: can it be done or is the trypanosome truly the ultimate immune destroyer and escape artist? Hum Vaccin. 2011; 7: 1225–1233. 10.4161/hv.7.11.18203 22205439 PMC3323498

[3] Delespaux V, De Koning HP. Drugs and drug resistance in African trypanosomiasis. Drug Resist Updat. 2007; 10: 30–50. 17409013 10.1016/j.drup.2007.02.004

[4] Stevenson P, Sones KR, Gicheru MM, Mwangi EK. Comparison of isometamidium chloride and homidium bromide as prophylactic drugs for trypanosomiasis in cattle at Nguruman, Kenya. Acta Trop. 1995; 59: 77–84. 7676909 10.1016/0001-706x(94)00080-k

[5] Holmes PH, Eisler MC, Geerts S. Current chemotherapy of animal trypanosomiasis In: Maudlin I, Holmes PH, Miles MA, editors. The trypanosomiases. Wallingford: CABI; 2004 pp. 431–44. ISBN 0-85199-475-X

[6] Tumir L-M, Radić Stojković M, Piantanida I. Come-back of phenanthridine and phenanthridinium derivatives in the 21st century. Beilstein J Org Chem. 2014; 10: 2930–2954. 10.3762/bjoc.10.312 25550761 PMC4273281

[7] Mulugeta W, Wilkes J, Mulatu W, Majiwa PA, Masake R, Peregrine AS. Long-term occurrence of *Trypanosoma congolense* resistant to diminazene, isometamidium and homidium in cattle at Ghibe, Ethiopia. Acta Trop. 1997; 64: 205–217. 9107367 10.1016/s0001-706x(96)00645-6

[8] Murilla GA, Peregrine AS, Ndung’u JM, Holmes PH, Eisler MC. The effects of drug-sensitive and drug-resistant *Trypanosoma congolense* infections on the pharmacokinetics of homidium in Boran cattle. Acta Trop. 2002; 81: 185–195. 11835895 10.1016/s0001-706x(01)00209-1

[9] Dagnachew S, Terefe G, Abebe G, Barry D, McCulloch R, Goddeeris B. In vivo experimental drug resistance study in *Trypanosoma vivax* isolates from tsetse infested and non-tsetse infested areas of Northwest Ethiopia. Acta Trop. 2015; 146: 95–100. 10.1016/j.actatropica.2015.03.014 25792418 PMC7612308

[10] Delespaux V, Vitouley HS, Marcotty T, Speybroeck N, Berkvens D, Roy K, et al Chemosensitization of *Trypanosoma congolense* strains resistant to isometamidium chloride by tetracyclines and enrofloxacin. PLoS Negl Trop Dis. 2010; 4: e828 10.1371/journal.pntd.0000828 20927189 PMC2946901

[11] Mungube EO, Vitouley HS, Allegye-Cudjoe E, Diall O, Boucoum Z, Diarra B, et al Detection of multiple drug-resistant *Trypanosoma congolense* populations in village cattle of south-east Mali. Parasit Vectors. 2012; 5: 155 10.1186/1756-3305-5-155 22852796 PMC3432589

[12] Tewelde N, Abebe G, Eisler M, McDermott J, Greiner M, Afework Y, et al Application of field methods to assess isometamidium resistance of trypanosomes in cattle in western Ethiopia. Acta Trop. 2004; 90: 163–170. 15177142 10.1016/j.actatropica.2003.11.012

[13] Delespaux V, Geysen D, Geerts S. Point mutations in mitochondrial topoisomerase enzymes of *Trypanosoma congolense* are not involved in isometamidium resistance. Mol Biochem Parasitol. 2007; 151: 137–140. 17123643 10.1016/j.molbiopara.2006.10.013

[14] De Koning HP, Anderson LF, Stewart M, Burchmore RJS, Wallace LJM, Barrett MP. The trypanocide diminazene aceturate is accumulated predominantly through the TbAT1 purine transporter: additional insights on diamidine resistance in African trypanosomes. Antimicrob Agents Chemother. 2004; 48: 1515–1519. 15105099 10.1128/AAC.48.5.1515-1519.2004 PMC400564

[15] Munday JC, Tagoe DNA, Eze AA, Krezdorn JAM, Rojas López KE, Alkhaldi AAM, et al Functional analysis of drug resistance-associated mutations in the Trypanosoma brucei adenosine transporter 1 (TbAT1) and the proposal of a structural model for the protein. Mol Microbiol. 2015; 96: 887–900. 10.1111/mmi.12979 25708978 PMC4755147

[16] Graf FE, Baker N, Munday JC, De Koning HP, Horn D, Mäser P. Chimerization at the AQP2-AQP3 locus is the genetic basis of melarsoprol-pentamidine cross-resistance in clinical *Trypanosoma brucei gambiense* isolates. Int J Parasitol Drugs drug Resist. 2015; 5: 65–68. 10.1016/j.ijpddr.2015.04.002 26042196 PMC4443405

[17] Delespaux V, Chitanga S, Geysen D, Goethals A, van den Bossche P, Geerts S. SSCP analysis of the P2 purine transporter TcoAT1 gene of *Trypanosoma congolense* leads to a simple PCR-RFLP test allowing the rapid identification of diminazene resistant stocks. Acta Trop. 2006; 100: 96–102. 17083909 10.1016/j.actatropica.2006.10.001

[18] Delespaux Vincent, De Koning HP. Transporters in antiparasitic drug development and resistance In: Jäger T, Koch O, Flohe L, editor. Trypanosomatid Diseases: Molecular Routes to Drug Discovery. Wiley-Blackwell; 2013 pp. 335–349. ISBN 978-3-527-33255-7

[19] Munday JC, Rojas López KE, Eze AA, Delespaux V, Van Den Abbeele J, Rowan T, et al Functional expression of TcoAT1 reveals it to be a P1-type nucleoside transporter with no capacity for diminazene uptake. Int J Parasitol Drugs drug Resist. 2013; 3: 69–76. 10.1016/j.ijpddr.2013.01.004 24533295 PMC3862423

[20] Roy Chowdhury A, Bakshi R, Wang J, Yildirir G, Liu B, Pappas-Brown V, et al The killing of African trypanosomes by ethidium bromide. PLoS Pathog. 2010; 6: e1001226 10.1371/journal.ppat.1001226 21187912 PMC3002999

[21] Schnaufer A, Clark-Walker GD, Steinberg AG, Stuart K. The F_1_-ATP synthase complex in bloodstream stage trypanosomes has an unusual and essential function. EMBO J. 2005; 24: 4029–4040. 16270030 10.1038/sj.emboj.7600862 PMC1356303

[22] Lukes J, Hashimi H, Zíková A. Unexplained complexity of the mitochondrial genome and transcriptome in kinetoplastid flagellates. Curr Genet. 2005; 48: 277–299. 16215758 10.1007/s00294-005-0027-0

[23] Michels PA, Bringaud F, Herman M, Hannaert V. Metabolic functions of glycosomes in trypanosomatids. Biochim Biophys Acta. 2006; 1763: 1463–1477. 17023066 10.1016/j.bbamcr.2006.08.019

[24] Alkhaldi AAM, Martinek J, Panicucci B, Dardonville C, Zíková A, De Koning HP. Trypanocidal action of bisphosphonium salts through a mitochondrial target in bloodstream form *Trypanosoma brucei*. Int J Parasitol Drugs Drug Resist. 2016; 6: 23–34. 10.1016/j.ijpddr.2015.12.002 27054061 PMC4805778

[25] Verner Z, Basu S, Benz C, Dixit S, Dobáková E, Faktorová D, et al Malleable mitochondrion of *Trypanosoma brucei*. Int Rev Cell Mol Biol. 2015; 315: 73–151. 10.1016/bs.ircmb.2014.11.001 25708462

[26] Clarkson AB Jr, Bienen EJ, Pollakis G, Grady RW. Respiration of bloodstream forms of the parasite *Trypanosoma brucei brucei* is dependent on a plant-like alternative oxidase. J Biol Chem. 1989; 264: 17770–17776. 2808350

[27] Chaudhuri M, Ott RD, Hill GC. Trypanosome alternative oxidase: from molecule to function. Trends Parasitol. 2006; 22: 484–491. 16920028 10.1016/j.pt.2006.08.007

[28] Roldán A, Comini MA, Crispo M, Krauth-Siegel RL. Lipoamide dehydrogenase is essential for both bloodstream and procyclic *Trypanosoma brucei*. Mol Microbiol. 2011; 81: 623–639. 10.1111/j.1365-2958.2011.07721.x 21631607

[29] Stephens JL, Lee SH, Paul KS, Englund PT. Mitochondrial fatty acid synthesis in *Trypanosoma brucei*. J Biol Chem. 2007; 282: 4427–36. 17166831 10.1074/jbc.M609037200

[30] Mazet M, Morand P, Biran M, Bouyssou G, Courtois P, Daulouède S, et al Revisiting the central metabolism of the bloodstream forms of *Trypanosoma brucei*: production of acetate in the mitochondrion is essential for parasite viability. PLoS Negl Trop Dis. 2013; 7: e2587 10.1371/journal.pntd.0002587 24367711 PMC3868518

[31] Basu S, Horáková E, Lukeš J. Iron-associated biology of *Trypanosoma brucei*. Biochim Biophys Acta. 2016; 1860: 363–370. 10.1016/j.bbagen.2015.10.027 26523873

[32] Shapiro TA, Englund PT. Selective cleavage of kinetoplast DNA minicircles promoted by antitrypanosomal drugs. Proc Natl Acad Sci U S A. 1990; 87: 950–954. 2153980 10.1073/pnas.87.3.950 PMC53387

[33] Gould MK, Schnaufer A. Independence from kinetoplast DNA maintenance and expression is associated with multidrug resistance in *Trypanosoma brucei* in vitro. Antimicrob Agents Chemother. 2014; 58: 2925–2928. 10.1128/AAC.00122-14 24550326 PMC3993240

[34] Boibessot I, Turner CMR, Watson DG, Goldie E, Connel G, McIntosh A., et al Metabolism and distribution of phenanthridine trypanocides in *Trypanosoma brucei*. Acta Trop. 2002; 84: 219–228. 12443800 10.1016/s0001-706x(02)00188-2

[35] Ibrahim HMS, Al-Salabi MI, El Sabbagh N, Quashie NB, Alkhaldi AAM, Escale R, et al Symmetrical choline-derived dications display strong anti-kinetoplastid activity. J Antimicrob Chemother. 2011; 66: 111–125. 10.1093/jac/dkq401 21078603 PMC3001849

[36] Lanteri CA, Tidwell RR, Meshnick SR. The mitochondrion is a site of trypanocidal action of the aromatic diamidine DB75 in bloodstream forms of Trypanosoma brucei. Antimicrob Agents Chemother. 2008; 52: 875–882. 18086841 10.1128/AAC.00642-07 PMC2258549

[37] Wilkes JM, Mulugeta W, Wells C, Peregrine AS. Modulation of mitochondrial electrical potential: a candidate mechanism for drug resistance in African trypanosomes. Biochem J. 1997; 326: 755–761. 9307025 10.1042/bj3260755 PMC1218730

[38] Sutherland IA, Peregrine AS, Lonsdale-Eccles JD, Holmes PH. Reduced accumulation of isometamidium by drug-resistant *Trypanosoma congolense*. Parasitology. 1991; 103: 245–251. 1745550 10.1017/s0031182000059527

[39] Sutherland IA, Mounsey A, Eisler M, Holmes PH. Kinetic modelling of isometamidium chloride (Samorin) uptake by *Trypanosoma congolense*. Parasitology. 1992; 105: 91–95. 1437280 10.1017/s0031182000073728

[40] Gudin S, Quashie NB, Candlish D, Al-Salabi MI, Jarvis SM, Ranford-Cartwright LC, et al *Trypanosoma brucei*: a survey of pyrimidine transport activities. Exp Parasitol. 2006; 114: 118–125. 16620810 10.1016/j.exppara.2006.02.018

[41] Teka IA, Kazibwe AJN, El-Sabbagh N, Al-Salabi MI, Ward CP, Eze AA, et al The diamidine diminazene aceturate is a substrate for the high-affinity pentamidine transporter: implications for the development of high resistance levels in trypanosomes. Mol Pharmacol. 2011; 80: 110–116. 10.1124/mol.111.071555 21436312 PMC3127539

[42] Ali JAM, Creek DJ, Burgess K, Allison HC, Field MC, Mäser P, et al Pyrimidine salvage in *Trypanosoma brucei* bloodstream forms and the trypanocidal action of halogenated pyrimidines. Mol Pharmacol. 2013; 83: 439–453. 10.1124/mol.112.082321 23188714 PMC4857052

[43] Figarella K, Uzcategui NL, Beck A, Schoenfeld C, Kubata BK, Lang F, et al Prostaglandin-induced programmed cell death in Trypanosoma brucei involves oxidative stress. Cell Death Differ. 2006; 13: 1802–1814. 16456581 10.1038/sj.cdd.4401862

[44] Gould MK, Vu XL, Seebeck T, De Koning HP. Propidium iodide-based methods for monitoring drug action in the kinetoplastidae: comparison with the Alamar Blue assay. Anal Biochem. 2008; 382: 87–93. 10.1016/j.ab.2008.07.036 18722997

[45] Wallace LJM, Candlish D, De Koning HP. Different substrate recognition motifs of human and trypanosome nucleobase transporters. Selective uptake of purine antimetabolites. J Biol Chem. 2002; 277: 26149–26156. 12004061 10.1074/jbc.M202835200

[46] Munday JC, Eze AA, Baker N, Glover L, Clucas C, Aguinaga Andrés D, et al *Trypanosoma brucei* aquaglyceroporin 2 is a high-affinity transporter for pentamidine and melaminophenyl arsenic drugs and the main genetic determinant of resistance to these drugs. J Antimicrob Chemother. 2014; 69: 651–663. 10.1093/jac/dkt442 24235095 PMC3922157

[47] Wilkes JM, Peregrine AS, Zilberstein D. The accumulation and compartmentalization of isometamidium chloride in *Trypanosoma congolense*, monitored by its intrinsic fluorescence. Biochem J. 1995; 312: 319–327. 7492332 10.1042/bj3120319 PMC1136262

[48] Ward CP, Burgess KE, Burchmore RJ, Barrett MP, De Koning HP. A fluorescence-based assay for the uptake of CPD0801 (DB829) by African trypanosomes. Mol Biochem Parasitol. 2010; 174: 145–149. 10.1016/j.molbiopara.2010.07.002 20637807

[49] Ward CP, Wong PE, Burchmore RJ, De Koning HP, Barrett MP. Trypanocidal furamidine analogues: influence of pyridine nitrogens on trypanocidal activity, transport kinetics, and resistance patterns. Antimicrob Agents Chemother. 2011; 55: 2352–2361. 10.1128/AAC.01551-10 21402852 PMC3088251

[50] Boyer PD. The ATP synthase-a splendid molecular machine. Annu Rev Biochem. 1997; 66: 717–749. 9242922 10.1146/annurev.biochem.66.1.717

[51] Dean S, Gould MK, Dewar CE, Schnaufer AC. Single point mutations in ATP synthase compensate for mitochondrial genome loss in trypanosomes. Proc Natl Acad Sci U S A. 2013; 110: 14741–14746. 10.1073/pnas.1305404110 23959897 PMC3767566

[52] Lai D-H, Hashimi H, Lun Z-R, Ayala FJ, Lukes J. Adaptations of *Trypanosoma brucei* to gradual loss of kinetoplast DNA: *Trypanosoma equiperdum* and *Trypanosoma evansi* are petite mutants of *T*. *brucei*. Proc Natl Acad Sci U S A. 2008; 105: 1999–2004. 10.1073/pnas.0711799105 18245376 PMC2538871

[53] Carnes J, Anupama A, Balmer O, Jackson A, Lewis M, Brown R, et al Genome and phylogenetic analyses of *Trypanosoma evansi* reveal extensive similarity to *T*. *brucei* and multiple independent origins for dyskinetoplasty. PloS Negl Trop Dis. 2015; 9: e3404 10.1371/journal.pntd.0003404 25568942 PMC4288722

[54] Eisler MC, Maruta J, Nqindi J, Connor RJ, UshewokunzeObatolu U, Holmes PH, et al Isometamidium concentrations in the sera of cattle maintained under a chemoprophylactic regime in a tsetse-infested area of Zimbabwe. Trop Med Int Health. 1996; 1: 535–541. 8765463 10.1046/j.1365-3156.1996.d01-87.x

[55] Sutcliffe OB, Skellern GG, Araya F, Cannavan A, Sasanya JJ, Dungu B, et al Animal trypanosomosis: making quality control of trypanocidal drugs possible. Rev Sci Tech. 2014; 33: 813–830. 25812206 10.20506/rst.33.3.2320

[56] Bridges DJ, Gould MK, Nerima B, Mäser P, Burchmore RJS, De Koning HP. Loss of the high-affinity pentamidine transporter is responsible for high levels of cross-resistance between arsenical and diamidine drugs in African trypanosomes. Mol Pharmacol. 2007; 71: 1098–1108. 17234896 10.1124/mol.106.031351

[57] Vincent IM, Creek D, Watson DG, Kamleh MA, Woods DJ, Wong PE, et al A molecular mechanism for eflornithine resistance in African trypanosomes. PLoS Pathog. 2010; 6: e1001204 10.1371/journal.ppat.1001204 21124824 PMC2991269

[58] Manzano JI, Lecerf-Schmidt F, Lespinasse M-A, Di Pietro A, Castanys S, Boumendjel A, et al Identification of specific reversal agents for Leishmania ABCI4-mediated antimony resistance by flavonoid and trolox derivative screening. J Antimicrob Chemother. 2014; 69: 664–672. 10.1093/jac/dkt407 24126793

[59] Antoniel M, Giorgio V, Fogolari F, Glick GD, Bernardi P, Lippe G. The oligomycin-sensitivity conferring protein of mitochondrial ATP synthase: emerging new roles in mitochondrial pathophysiology. Int J Mol Sci. 2014; 15: 7513–7536. 10.3390/ijms15057513 24786291 PMC4057687

[60] Baker N, Hamilton G, Wilkes JM, Hutchinson S, Barrett MP, Horn D. Vacuolar ATPase depletion affects mitochondrial ATPase function, kinetoplast dependency, and drug sensitivity in trypanosomes. Proc Natl Acad Sci U S A. 2015; 112: 9112–9117. 10.1073/pnas.1505411112 26150481 PMC4517229

[61] Huang G, Ulrich PN, Storey M, Johnson D, Tischer J, Tovar JA, et al Proteomic analysis of the acidocalcisome, an organelle conserved from bacteria to human cells. PLoS Pathog. 2014; 10:e1004555 10.1371/journal.ppat.1004555 25503798 PMC4263762

[62] Mäser P, Sütterlin C, Kralli A, Kaminsky R. A nucleoside transporter from *Trypanosoma brucei* involved in drug resistance. Science. 1999; 285: 242–244. 10398598 10.1126/science.285.5425.242

