# Supplementary figures and images for: Reduced Mitochondrial Membrane Potential Is a Late Adaptation of Trypanosoma brucei brucei to Isometamidium Preceded by Mutations in the γ Subunit of the F1Fo-ATPase
Source: PLoS Negl Trop Dis. 2016 Aug 12;10(8):e0004791. doi: 10.1371/journal.pntd.0004791 (PMC4982688; doi:10.1371/journal.pntd.0004791)

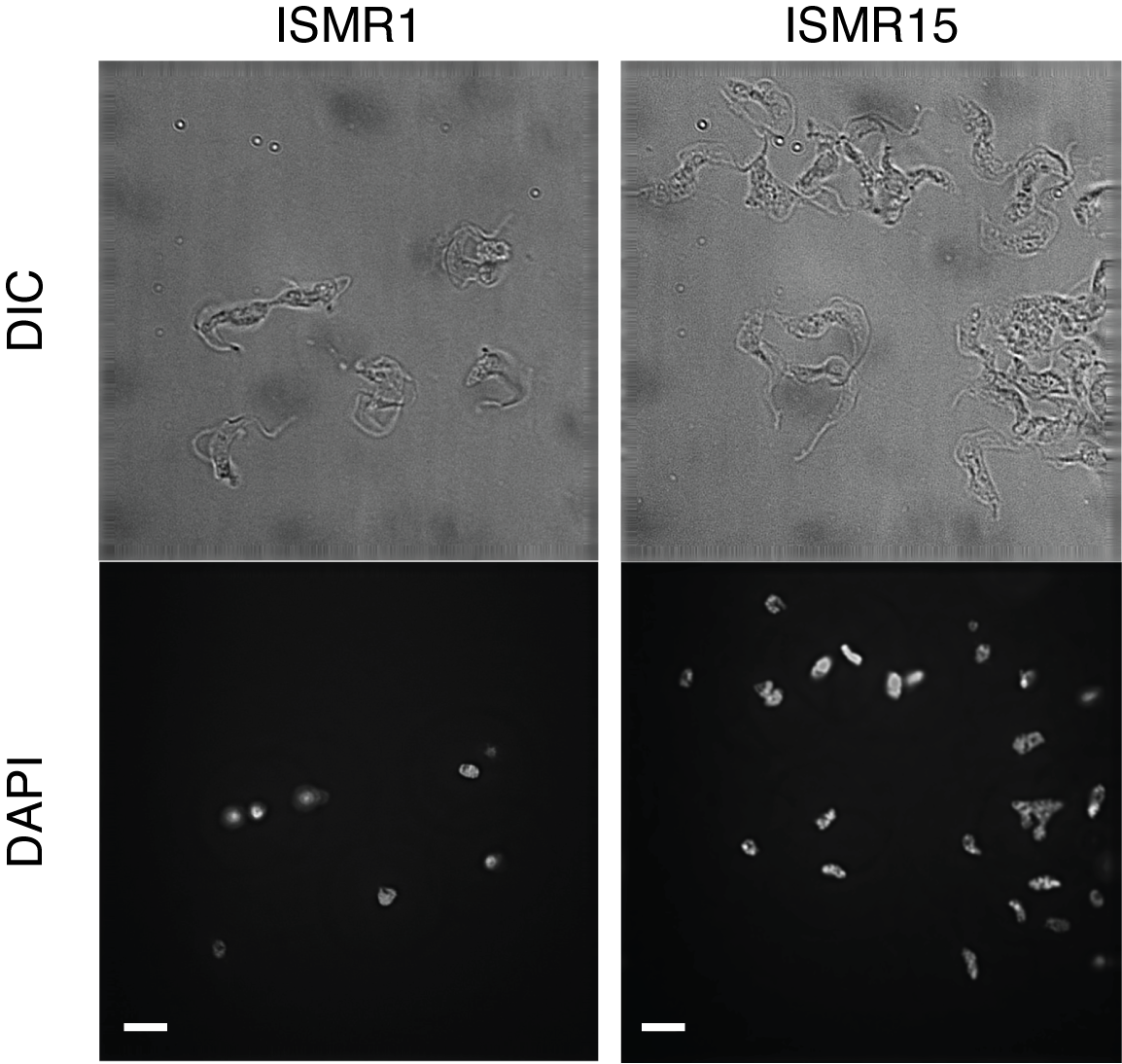

Supplement: S1 Fig — None of the cells in the population contain a kinetoplast. (TIF) [file pntd.0004791.s001.tif]

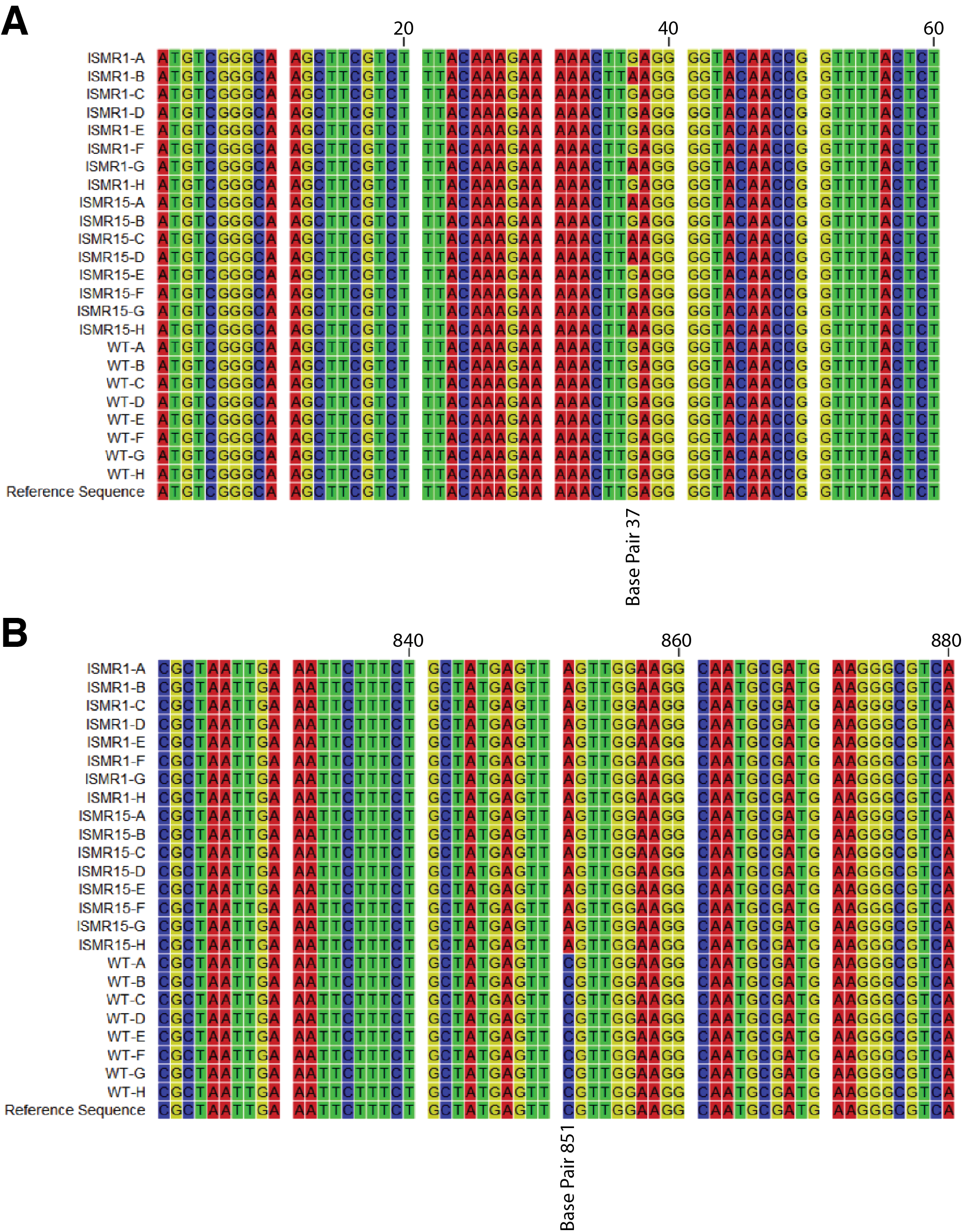

Supplement: S2 Fig — The full length ORF of subunit γ was amplified using a proofreading polymerase. The products were A-tailed using Taq polymerase and cloned into the pGEM-T Easy vector. Eight clones from each trypanosome cell line were sequenced and aligned; the sequence for strain 427 was taken from the TriTryp genome database (www.tritrypdb.org) and used as a reference sequence for all alignments. (A) Sequence of nucleotides 1–60. (B) Sequence of nucleotides 820–880. (TIF) [file pntd.0004791.s002.tif]

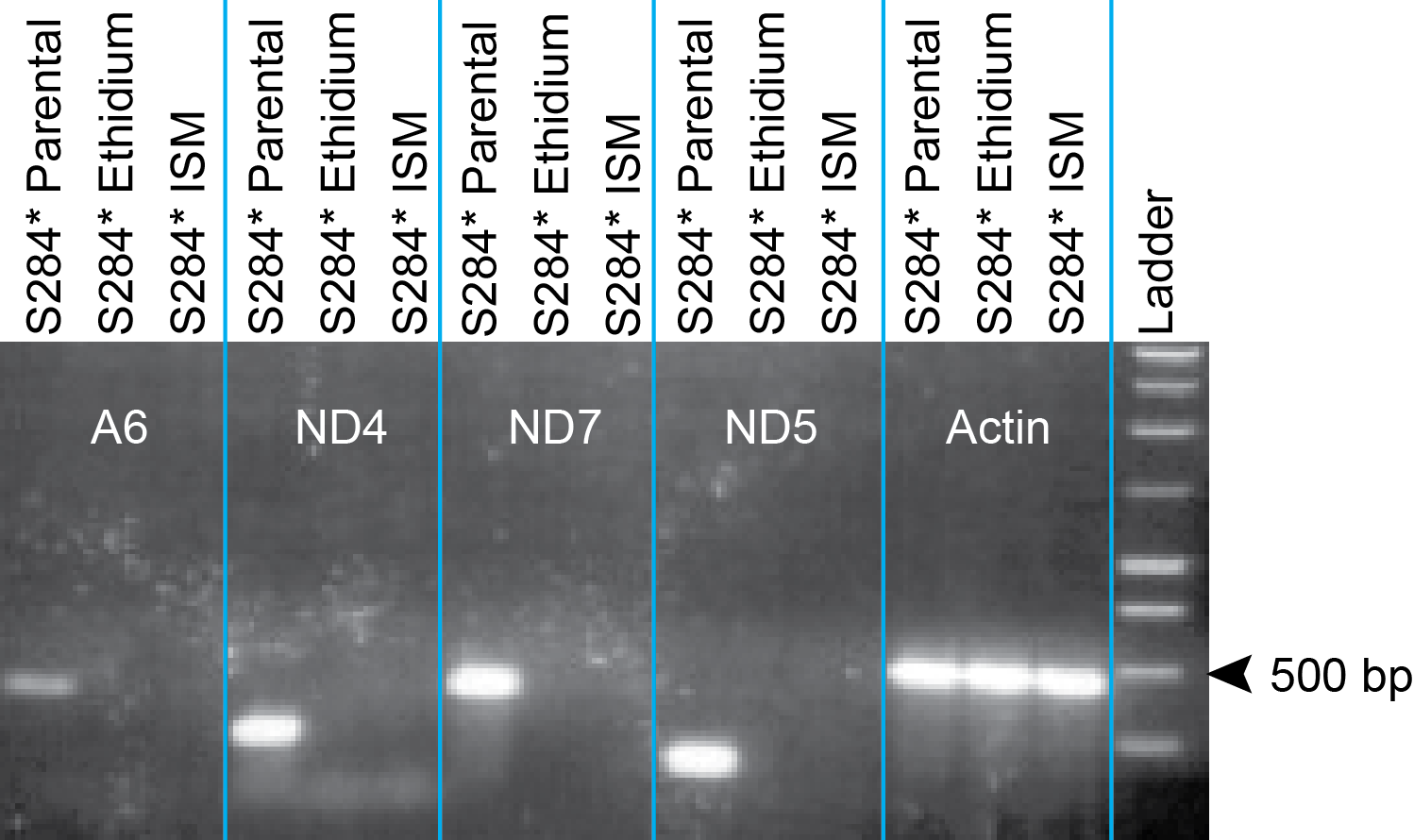

Supplement: S3 Fig — Genomic DNA was extracted from Tb427WT trypanosomes with one allele of ATP synthase subunit γ endogenously replaced with a version containing the S284* mutation, as well as from the same strain after 7 days exposure to either 20 nM ISM or ethidium bromide. PCR amplification was carried out using primers specific for the genes stated (S1 Table). (TIF) [file pntd.0004791.s003.tif]
