# Supplementary material for: Reduced Mitochondrial Membrane Potential Is a Late Adaptation of Trypanosoma brucei brucei to Isometamidium Preceded by Mutations in the γ Subunit of the F1Fo-ATPase
Source: PLoS Negl Trop Dis. 2016 Aug 12;10(8):e0004791. doi: 10.1371/journal.pntd.0004791 (PMC4982688; doi:10.1371/journal.pntd.0004791)
Supplement: S1 Table — (DOCX) [file pntd.0004791.s004.docx]

**S1 Table.** Oligonucleotide primer sequences used in this study.

| **Primer for:** | **Nucleotide sequence** | **Product size:** |
| --- | --- | --- |
| A6 | Fwd: 5’-AAAAATAAGTATTTTGATATTATTAAAG-3’  Rev: 5’-TATTATTAACTTATTTGATC-3’ | 381 bp |
| ND4 | Fwd: 5’-TGTGTGACTACCAGAGAT-3’  Rev: 5’-ATCCTATACCCGTGTGTA-3’ | 256 bp |
| ND5 | Fwd: 5’-TGGGTTTATATCAGGTTCATTTATG-3’  Rev: 5’-CCCTAATAATCTCATCCGCAGTACG-3’ | 395 bp |
| ND7 | Fwd: 5’-ATGACTACATGATAAGTA-3’  Rev: 5’-CGGAAGACATTGTTCTACAC-3’ | 161 bp |
| Actin | Fwd: 5’-CCGAGTCACACAACGT-3’  Rev: 5’-CCACCTGCATAACATTG-3’ | 456 bp |
| Minicircle Type A | Fwd: 5’-GGGTTTTTTAGGTCCGAG-3’  Rev: 5’-CCGAAAATAGCACGTG-3’ | 1001 bp |
| TbAT-1 | Fwd: 5’-CGTCACATCTTTCTTCTCG-3’  Rev: 5’-CTTGGGAAGCCCCTCATTGACAGCC-3’ | 889 bp |
| ATP synthase γ | For: 5’-GCTTACACTATTGCTTTACTGCG-3’  Rev: 5’-CGGTCGCACGCATACCTACC-3’ | 1045 bp |

List of Forward (Fwd) and reverse (Rev) PCR primers used in this study, together with the predicted products and product sizes.
